# Late morfofunctional alterations of the Sertoli cell caused by doxorubicin administered to prepubertal rats

**DOI:** 10.1186/1477-7827-10-79

**Published:** 2012-09-11

**Authors:** Otávio Brilhante, Fatima K Okada, Estela Sasso-Cerri, Taiza Stumpp, Sandra M Miraglia

**Affiliations:** 1 Centre for Health and Rural Technology, Academic Unit of Veterinary Medicine, Federal University of Campina Grande, Patos, Paraíba, Brazil; 2 Department of Morphology and Genetics, Developmental Biology Laboratory, Federal University of São Paulo. Vila Clementino, São Paulo, SP, Brazil; 3 Department of Morphology, Laboratory of Histology and Embryology, Dental School of São Paulo State University (UNESP), Araraquara, SP, Brazil

**Keywords:** Doxorubicin, Sertoli cell, Spermatogenesis, Rat, Transferrin

## Abstract

**Background:**

Doxorubicin is a potent chemotherapeutic drug used against a variety of cancers. It acts through interaction with polymerases and topoisomerase II and free radical production. Doxorubicin activity is not specific to cancer cells and can also damage healthy cells, especially those undergoing rapid proliferation, such as spermatogonia. In previous studies our group showed that etoposide, another topoisomarese II poison, causes irreversible damage to Sertoli cells. Thus, the aim of this study was to address the effects of doxorubicin on Sertoli cell morphology and function and on the seminiferous epithelium cycle when administered to prepubertal rats.

**Methods:**

Prepubertal rats received the dose of 5 mg/Kg of doxorubicin, which was fractioned in two doses: 3 mg/Kg at 15dpp and 2 mg/Kg at 22dpp. The testes were collected at 40, 64 and 127dpp, fixed in Bouin’s liquid and submitted to transferrin immunolabeling for Sertoli cell function analysis. Sertoli cell morphology and the frequency of the stages of the seminiferous epithelium cycle were analyzed in PAS + H-stained sections.

**Results:**

The rats treated with doxorubicin showed reduction of transferrin labeling in the seminiferous epithelium at 40 and 64dpp, suggesting that Sertoli cell function is altered in these rats. All doxorubicin-treated rats showed sloughing and morphological alterations of Sertoli cells. The frequency of the stages of the seminiferous epithelium cycle was also affected in all doxorubicin-treated rats.

**Conclusions and discussion:**

These data show that doxorubicin administration during prepuberty causes functional and morphological late damage to Sertoli cells; such damage is secondary to the germ cell primary injury and contributed to enhance the spermatogenic harm caused by this drug. However, additional studies are required to clarify if there is also a direct effect of doxorubicin on Sertoli cells producing a primary damage on these cells.

## Background

Doxorubicin is an anthracyclic antibiotic with potent chemotherapeutic activity against a variety of cancers. The toxicity of this drug is mediated by its interaction with topoisomerase II, an enzyme that is abundant in cells undergoing rapid and constant proliferation. Doxorubicin toxicity can also be mediated by the generation of free radicals [1,2] and lipid peroxidation [3]. Both mechanisms are not specific to cancer cells and can damage healthy cells [4-7]. In the testis, spermatogonia are the main doxorubicin target, as observed after etoposide treatment, because of their intense and continuous proliferative activity [8]. The administration of doxorubicin to prepubertal rats causes damage to spermatogonia [9] and irreversible damages to adult spermatogenesis [9,10]. Extensive injuries such as decrease of spermatogonia, degeneration and/or decrease of early spermatocytes, vacuolated seminiferous epithelium, reduction of epididymis cauda sperm count and sperm motility, which were caused by a single dose of doxorubicin (10 mg/Kg bw), have been characterized in the rat testicular tissue after the schedule’s termination [11].

Other chemotherapeutic drugs, such as cisplatin [12,13] and etoposide [14-16], another topoisomerase-interacting drug, also cause damage to germ cells, especially when administered during prepubertal and peripubertal phases. In previous studies our group showed that etoposide administration to prepubertal rats causes irreversible damage to Sertoli cells [17,18], leading to severe spermatogenesis impairment.

Sertoli cells play a key role in spermatogenesis control and germ cell development [19,20] and are pivotal for testicular homeostasis; this phenomenon is maintained through the complex interactions with germ cells and with the other testicular somatic cells, such as Leydig and myoid cells [21]. Sertoli cell alterations can cause severe damage to spermatogenesis [22-24]. Some Sertoli cell toxicants, such as 2,5-Hexanedione [25] and Mono-(2-ethylhexyl) Phthalate [26], have been shown to cause massive germ cell death. On the other hand, germ cell death can also lead to functional and morphological alterations of Sertoli cells [27]. Because Sertoli cells are considered the most resistant cell in the testis, most of the alterations caused to these cells by the administration of different toxicants are considered to be secondary effects of germ cell death [28,29]. However, previous studies have suggested that germ cell death caused by etoposide administration to prepubertal rats is not the unique factor responsible for the damages observed in Sertoli cells, since even after seminiferous epithelium recovery, Sertoli cells still showed functional and morphological alterations [17,18]. The transferrin is vital to the regulation of spermatogenesis. It has been demonstrated that transferrin constitutes 5% of all proteins secreted by Sertoli cell [30]. In addition, it is a reliable instrument of investigation of the Sertoli cell function, both in vitro and in vivo [31,32] and it has been considered one of the best markers for this scope [33]. Consequently, transferrin has been largely utilized for the evaluation of the Sertoli cell function and its labeling has been successfully included in studies that aimed to investigate the possible functional alterations produced as a response to harmful events [34,35]. Germ cells also express transferrin receptors [36]. Therefore, transferrin labeling was utilized in the current study.

The studies performed by our group have suggested that prepubertal testes are more sensitive to chemotherapeutic drugs than adult testes [9,15,16]. Doxorubicin is largely used in child cancer treatments and has been shown to be very aggressive to germ cells. However, the effects of doxorubicin administration on the morphology and function of Sertoli cells have not been detailedly addressed yet, especially during prepuberty. Thus, the aim of the present study was to evaluate the effects of doxorubicin on Sertoli cell function and morphology when administered to prepubertal rats during key periods of testicular and Sertoli cell development.

## Methods

### Animals and drug administration

Sixty male Wistar rats (*Rattus norvegicus albinus*) were maintained under 12/12 hr light/dark cycles, at 21-23°C room temperature; standardized lab chow (Nuvilab CR1, Nuvital®, Curitiba, PR, Brazil) and water were provided *ad libitum*. The protocol regarding animal care and treatment was approved by the Ethical Committee for Animal Research of the Federal University of São Paulo, Brazil (reference number: 0559/08).

The animals were distributed into two major groups: control (C) and doxorubicin-treated (D). The rats from group D received 5 mg/Kg of doxorubicin (Rubidox®, Bergamo – São Paulo, Brazil) by intraperitoneal route. This dose was fractioned into two doses: one of 3 mg/Kg that was administered at 15 days postpartum (dpp) and one of 2 mg/Kg that was administered at 22dpp. The rats from the control group received 0.9% saline solution at the same ages and volume administered to the doxorubicin-treated group. The rats were maintained under standard conditions of luminosity (12 hr light/12 hr dark) and temperature (22-23°C). Food and water were allowed *ad libitum*.

The C and D groups were subdivided into three subgroups of 10 rats each, according to the ages of euthanasia: 40dpp (subgroups C40 and D40); 64dpp (subgroups C64 and D64) and 127dpp (subgroups C127 and D127). These ages were chosen because they represent key time points of spermatogenesis development, i.e., at 40dpp the rats are considered peripubertal [13], at 64dpp they have already reached puberty [37] but are not sexually mature and at 127dpp they are adults [14] and sexually mature [38].

### Testis collection and analysis

At the ages previously specified, the rats were weighed and submitted to euthanasia through CO_2_ inhalation according to recommendations of the Ethical Committee of the Federal University of Sao Paulo (UNIFESP). The testes were removed, weighed and had their volume measured according to the Scherle’s method [39]. Subsequently, the testes were immersion-fixed in Bouin’s liquid for 48 hr. Each testis was transversally cut and one half was embedded in Paraplast Plus® with DMSO (Sigma) and the other half was paraffin-embedded. From the fragments embedded in Paraplast Plus®, 3μm cross-sections were obtained and submitted to the Periodic Acid-Schiff histochemical method (PAS) and counterstained with Harris’s Hematoxilin (H). From the paraffin-embedded fragments, 7μm cross-sections were obtained and submitted to the transferrin immunolabeling, as described below.

### Transferrin immunolabeling

The sections obtained from the paraffin blocks were dewaxed, washed in running tap water for Bouin’s elimination and treated with 3% hydrogen peroxide for 15 minutes. The slides were washed in phosphate buffer (PBS, pH 7.2) and incubated with 7% BSA for 15 minutes. The slides were then incubated with the primary antibody anti-transferrin (1:1000, ICN Aurora, Ohio, USA) for 1 hr, washed in PBS and incubated with the secondary antibody (LSAB®, Dako, California, USA) for 30 minutes. After that, the slides were washed in PBS and incubated with Streptavidin-Peroxidase (LSAB®, Dako, California, USA) for 30 minutes. The reaction was revealed with DAB (DAKO, California, USA) and counterstained with Harris’s Haematoxylin. Negative control slides were performed by incubating the sections with non-immune serum.

### Stereology

To investigate transferrin production by Sertoli cells, the volume density (Vv) of transferrin-positive tissue in the seminiferous epithelium and in the interstitial tissue was obtained through the ratio between the positive tissue in each of these compartments and the total testicular tissue analyzed [17]. These measurements were obtained using a Leica QWin Analysis System (Leica - Cambridge, England) with a x20 objective lens.

### Histopathological analysis and frequency of the stages of the seminiferous epithelium cycle

For the histopathological analysis, the testicular sections from the Paraplast blocks were totally analyzed and the alterations, especially the Sertoli cell alterations, were described.

The frequencies of the stages of the seminiferous epithelium cycle were obtained from Parablast-embedded testes and the analysis was performed according to Hess et al. [40]. Two hundred seminiferous tubule cross-sections were analyzed per rat, which is the minimum number of sections that must be analyzed when the number of animals per group is 10 (n = 10) [32]. The identification of the stages of the seminiferous epithelium cycle was based on the classification of Leblond and Clermont [41]. The frequency of the stages, in percentage, was calculated by the ratio between the number of sections in each stage and the total number of analyzed sections multiplying by 100 [42]. Because the stages II and III as well as the stages XII and XIII are very similar between each other, they were grouped according to previously described [43-45]. This analysis was performed under a light microscope using x50 and x100 objective lenses.

### Statistical analysis

The data were submitted to *t* test. The results were considered significant when p ≤ 0.05.

## Results

### Testicular histopathology

**Figure 1:**
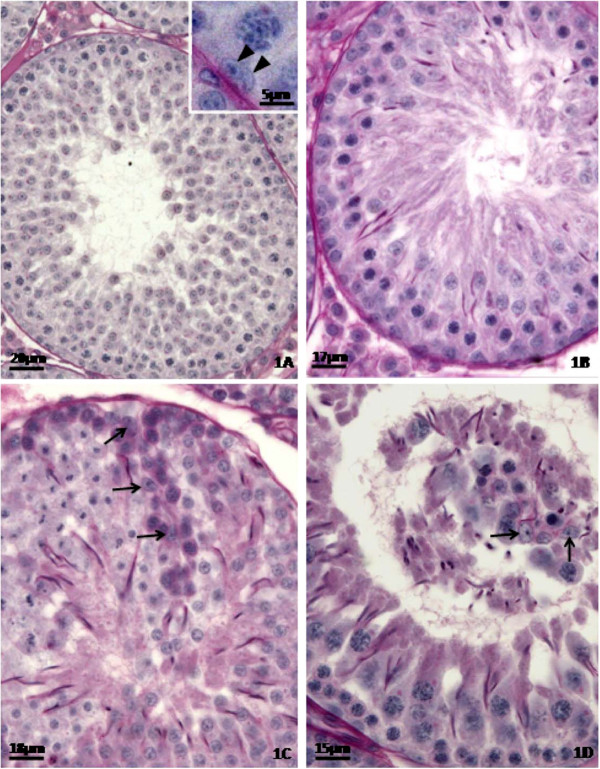
**Testicular cross-sections of 40 day-old control (1A) and doxorubicin-treated (1B-1D) rats submitted to PAS + H histochemical method.** The Figure 1**A** shows seminiferous epithelium presenting normal morphology. The nuclei of Sertoli cells show evident nucleoli and are located close to the basal membrane of the seminiferous epithelium (inset; arrowheads). In the Figure 1**B**, a tubular section showing germ cell depletion is observed. Figure 1**C** depicts a detached portion of seminiferous epithelium in which Sertoli cell nuclei are observed (arrows). The Figure 1**D** shows Sertoli cell nuclei into the tubular lumen (arrows).

**Figure 2:**
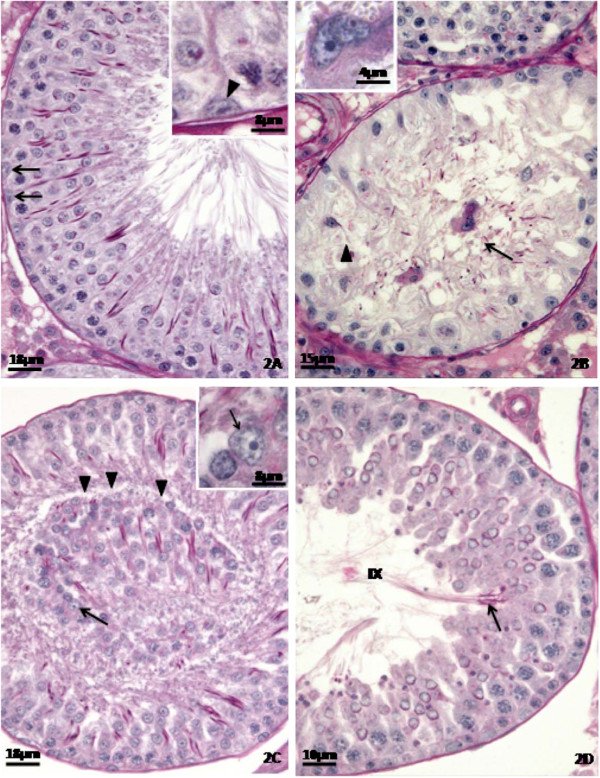
**Testicular cross-sections of 64 day-old control (Figure**2**A) and doxorubicin-treated (Figures**2**B-D) rats submitted to the PAS + H method.** The Figure 2**A** depicts part of a tubular section containing Sertoli cell nuclei with normal morphology. These nuclei are located close to the basal membrane (arrows) of the seminiferous epithelium and show evident nucleolus (inset; arrowhead). In the Figure 2**B**, a Sertoli cell only tubular section is observed. In this tubular section, one of the Sertoli cell nuclei is far from the basal membrane (arrowhead) and another is sloughed into the tubular lumen (arrow). The Figure 2**C** depicts a sloughed portion of seminiferous epithelium (arrowheads) into the tubular lumen in which a Sertoli cell nucleus can be seen (arrow). In the inset, this Sertoli cell nucleus with irregular profile shows abnormal clear areas (arrow). Figure 2**D** shows a tubular section in stage IX of the seminiferous epithelium cycle with retention of step 19 spermatids (arrow). Note the presence of intraepithelial spaces and free primary spermatocytes associated.

**Figure 3:**
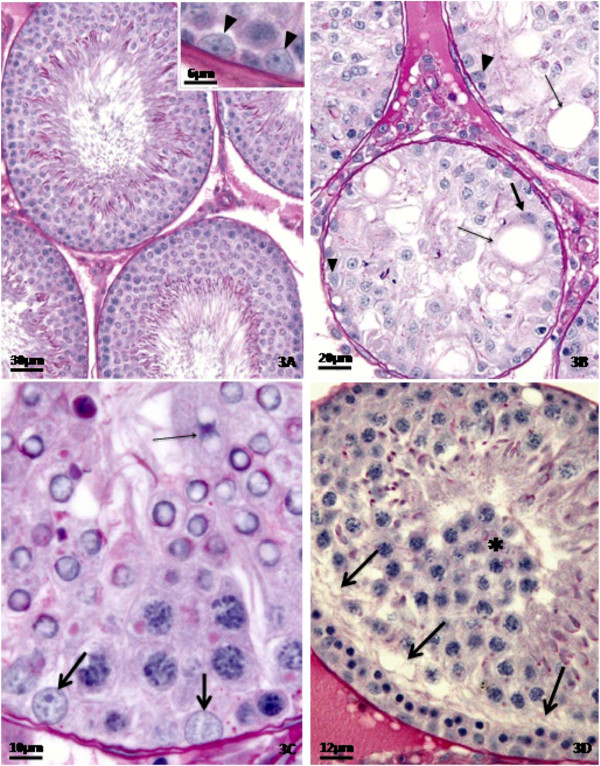
**Testicular cross-sections of 127 day-old control (Figure**3**A) and doxorubicin-treated (Figures**3**B-**3**D) rats submitted to the PAS + H method.** The Figure 3**A** depicts seminiferous tubule sections showing normal morphology. The Sertoli cells show normal nuclei (inset; arrowheads). In the Figure 3**B**, tubular sections with severe germ cell depletion and intraepithelial vacuoles (thin arrows) are seen. A displaced Sertoli cell nucleus from the basal membrane is also observed (thick arrow). Some Sertoli cell nuclei show abnormal profile (arrowheads). The Figure 3**C** shows Sertoli cell nuclei with abnormal morphology, showing round shape (thick arrows); in one of them the nucleolus is not evident. A binucleated formation of round spermatids can also be noted into the lumen (thin arrow). The Figure 3**D** depicts a portion of a seminiferous tubule showing a clear area without germ cells (arrows) and many primary spermatocytes in the tubular lumen (asterisk).

The histopathological analysis of the testes (Figures 1, 2 and 3) showed that the control rats presented normal morphology of seminiferous epithelium and of Sertoli cells at 40 (Figure 1A), 64 (Figure 2A) and 127 (Figure 3A) days of age. The rats treated with doxorubicin showed seminiferous epithelium vacuolization (Figure 3B) and germ cell depletion (Figures 1B, 2B and 3B–D). These alterations appeared at all ages, but became more intense at 64 days. Sertoli cell also showed morphological alterations in all doxorubicin-treated rats. Their nuclei showed abnormal morphology (Figures 2C and 3B–C) and some of them were distant from the basal membrane (Figures 1C, 2B, 3B) or even in the tubular lumen (Figures 1D and 2B–C). Eventually, Sertoli cell only tubular sections were observed (Figure 2B). The rats from D64 and D127 subgroups showed intense disorganization of the seminiferous epithelium and elongated spermatid retention at stage IX of the seminiferous epithelium cycle (Figure 2D). The rats from D127 subgroup showed partial recovery of spermatogenesis.

### Transferrin labeling and Sertoli cell function

**Figure 4:**
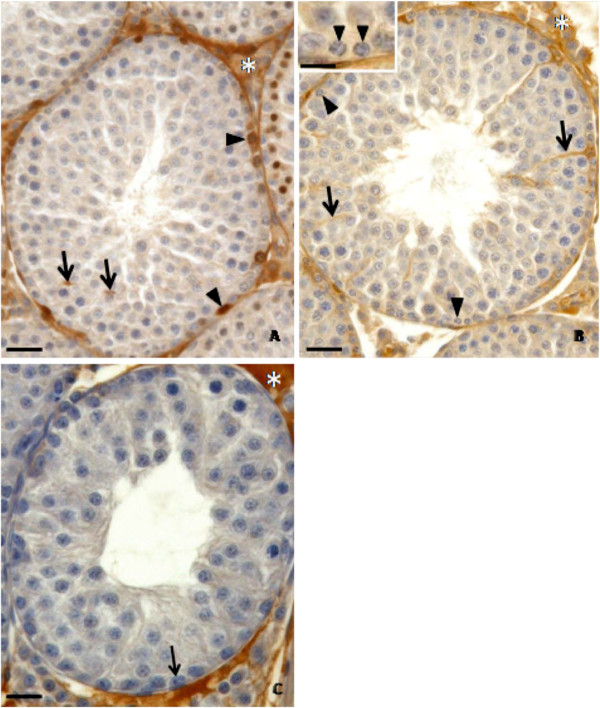
**Testicular cross-sections of 40 day-old control (Figure**4**A) and doxorubicin-treated (Figure**4**B-C) rats submitted to transferrin labeling.** The Figure 4**A** shows weak transferrin labeling in the Sertoli cell cytoplasm (arrows) of the control rat and strongly transferrin-positive germ cells, probably spermatogonia, which are located in the basis of the seminiferous epithelium (arrowheads). In the Figure 4**B** (doxorubicin-treated rat), although Sertoli cell cytoplasm is positive (arrows), no labeling is observed in the spermatogonia (arrowheads). The Figure 4**C** depicts a seminiferous tubule cross-section showing germ cell depletion and no transferrin labeling. Sertoli cell nucleus (arrow). Note the intense labeling in the interstitial tissue (Figures 4A-C; asterisks).

**Figure 5:**
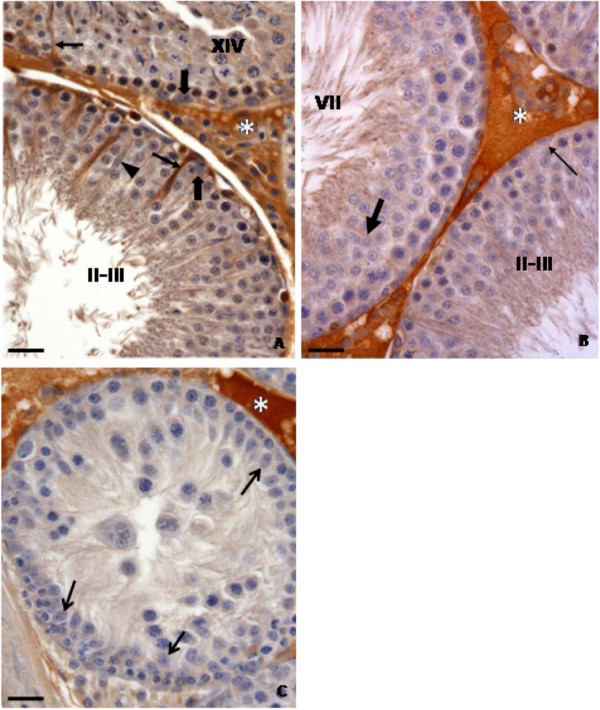
**Testicular cross-sections of 64 day-old control (Figure**5**A) and doxorubicin-treated (Figure**5**B-C) rats submitted to transferrin labeling.** In the Figure 5**A** (control rat), intense transferrin labeling is observed in the Sertoli cell cytoplasm (thin arrows) in both seminiferous tubule sections (stages II-III and XIV). Sertoli cell nuclei (thick arrows) and elongated spermatids (arrowhead) are not labeled. The Figure 5**B** (64 day-old doxorubicin-treated rat) shows very weak transferrin labeling in the Sertoli cell cytoplasm of tubules at stages II-III and VII (thick arrow); however, no labeling is observed in the Sertoli cell nucleus (thin arrow). The Figure 5**C** depicts a seminiferous tubule cross-section showing intense germ cell depletion in which no transferrin labeling is observed. Interstitial tissue immunolabeling is also noted (Figures 5A; asterisks).

**Figure 6:**
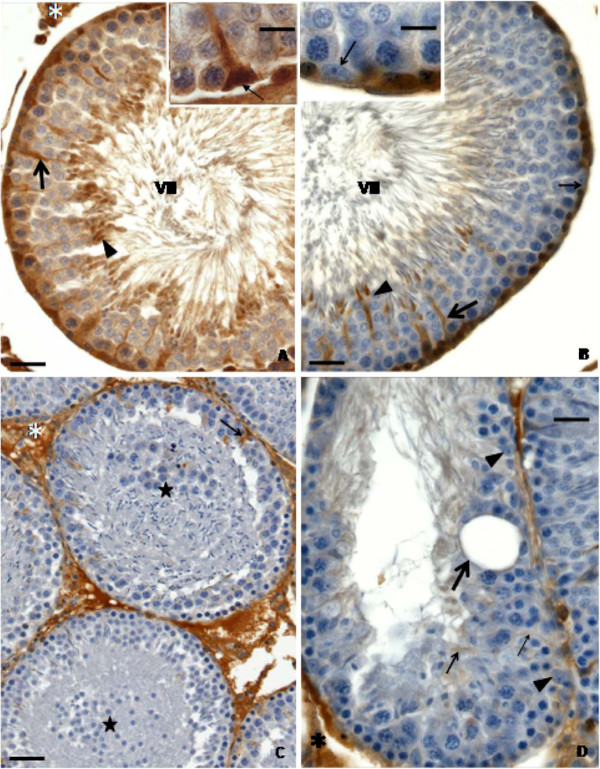
**Testicular cross-sections of 127 day-old control (Figure**6**A) and doxorubicin-treated (Figure**6**B-D) rats submitted to transferrin labeling.** The Figures 6**A**-**B** show intense transferrin labeling in the seminiferous epithelium (stage VII). However, in the Figure 6A (control group), the labeling is more abundant than in the Figure 6B (doxorubicin-treated group). In the control group (Figure 6A), transferrin labeling is observed in the Sertoli cell cytoplasm (thick arrow) and nucleus (inset, thin arrow) as well as in the elongated spermatids (arrowhead). In the doxorubicin-treated group (Figure 6B) transferrin labeling is observed in the Sertoli cell cytoplasm (thick arrow) and in the elongated spermatids (arrowhead), but not in the Sertoli cell nucleus (inset, thin arrow). The Figure 6**C** shows two seminiferous tubule cross-sections containing large portions of sloughed seminiferous epithelium (stars). In one of them, transferrin positive Sertoli cell cytoplasm is observed (arrow). In the Figure 6**D**, a tubular section with intense germ cell depletion shows weak Sertoli cell cytoplasm immunolabeling (thin arrows) and no labeling in the Sertoli cell nucleus (arrowheads). Intraepithelial vacuole (thick arrow). Note the intense labeling in the interstitial tissue (Figure 6A-D; asterisks).

Transferrin labeling observed in the seminiferous epithelium (Figures 4, 5 and 6) was dependent on the treatment (doxorubicin or saline solution) applied, on the euthanasia age and on the stage of the seminiferous epithelium cycle. The rats from C40 (Figure 4A) and D40 (Figures 4B-C) subgroups showed few labeled regions in the seminiferous epithelium. In the C40 subgroup, few Sertoli cells showed weak labeling in the cytoplasm (Figure 4A); however, cells in the basal region of seminiferous tubules, probably spermatogonia, showed intense labeling (Figure 4A). The D40 subgroup showed transferrin labeling in the Sertoli cells, but no labeling in the spermatogonia were observed (Figure 4B). Some hypotrophic tubular sections from D40 rats did not show any transferrin labeling (Figure 4C). On the other hand, C64 (Figure 5A) and C127 (Figure 6A) control subgroups showed strong and abundant labeling in the seminiferous epithelium. In these subgroups, Sertoli cells (Figures 5A, 6A) and elongated spermatids (Figure 6A) showed intense labeling. In both C64 and C127 subgroups, transferrin labeling was more abundant in stages II-III/XIV (Figure 5A) and VII (Figure 6A), respectively. In the D64 subgroup, some tubular sections in stages II-III and VII showed weak labeling in the Sertoli cell cytoplasm and no labeling in the nucleus (Figure 5B). In the seminiferous tubules with intense germ cell depletion, no transferrin labeling was observed (Figure 5C). In the D127 subgroup, intense transferrin labeling was observed in the Sertoli cell cytoplasm and in some germ cells located in the basal region of the seminiferous epithelium (Figure 6B), as observed in the C127 subgroup (Figure 6A); however, at the stage VII of seminiferous epithelium, the labeling seemed to be less abundant in the D127 subgroup than in the C127 subgroup (Figures 6A–B). In this subgroup (C127), transferrin labeling was observed either in the cytoplasm or nucleus of Sertoli cells (Figure 6A), while in D127, these cells showed only cytoplasm immunolabeling. Moreover, in some elongated spermatids, the strong transferrin labeling was also more abundant in C127 (Figure 6A) than in D127 (Figure 6B). In the D127 subgroup, some tubular sections with intense germ cell depletion showed rare transferrin immunolabeling (Figure 6C–D).

**Figure 7:**
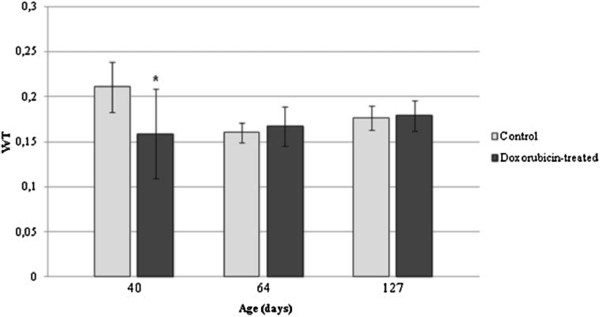
**Volume density of transferrin-positive total testicular tissue (VvT) in the control and doxorubicin-treated rats.** The VvT showed significant reduction in the 40dpp doxorubicin-treated rats (*) when compared with the corresponding control subgroup. No alteration of this parameter was observed in the 64 and 127dpp doxorubicin-treated rats.

**Figure 8:**
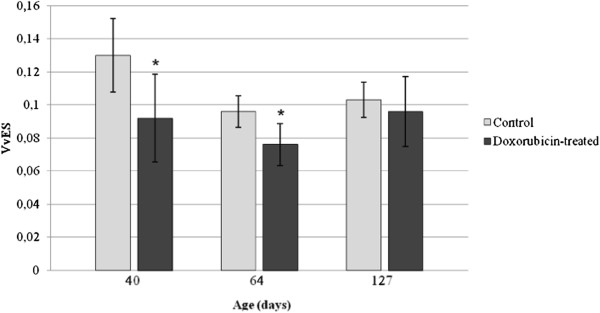
**Volume density of transferrin-positive seminiferous epithelium (VvES) in the control and doxorubicin-treated rats.** A significant reduction of this parameter was observed in the doxorubicin-treated rats at 40 and 64dpp (*) when compared with the corresponding control subgroups. The 127dpp doxorubicin-treated rats showed an important recovery of the VvES.

**Figure 9:**
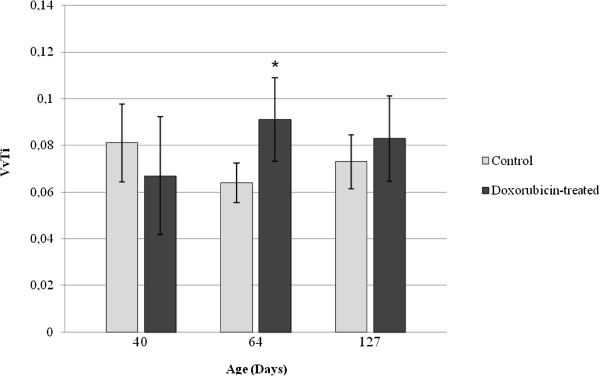
**Volume density of transferrin-positive interstitial tissue (VvTi) in the control and doxorubicin-treated rats.** Only the 64dpp doxorubicin-treated rats showed an increase of the VvTi (*) when compared with the control subgroup. Although the 40dpp doxorubicin-treated rats have shown a smaller mean value of this parameter and the 127dpp rats have shown a higher mean value of VvTi, these data were not significant.

The pattern of transferrin labeling (i.e., the stages in which transferrin labeling was observed and the cell types that were labeled) in the doxorubicin-treated rats was similar to that observed in the control rats. However, the volume density of transferrin-positive total testicular tissue reduced in the D40 subgroup (Figure 7) when compared to C40 subgroup, whereas in D64 and D127 this parameter was not altered in comparison to the corresponding control subgroups C64 and C127. On the other hand, in the D40 and D64 subgroups, the volume density of transferrin labeling in the seminiferous epithelium reduced when compared with the corresponding control subgroups C40 and C64 (Figure 8). No significant alteration of the volume density of transferrin labeling in the seminiferous epithelium was observed in the D127 subgroup in relation to C127 subgroup (Figure 8). The labeling pattern in the interstitial tissue was very similar in the control and doxorubicin-treated rats. Only the D64 subgroup showed volume density reduction of the transferrin-positive interstitial tissue (Figure 9).

### Frequency of the stages of the seminiferous epithelium cycle

**Figure 10:**
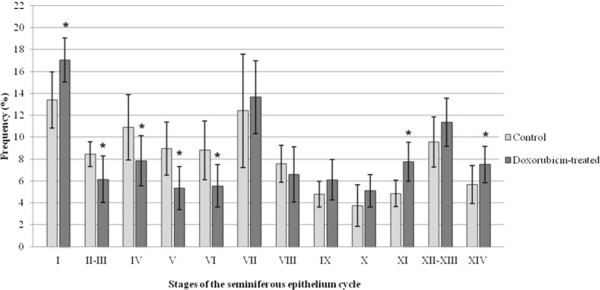
**Frequency of the stages of the seminiferous epithelium cycle in the 40-day-old rats.** An increase in the frequency of stages I, XI and XIV and a decrease of the frequency of stages II to VI were observed at this age. Statistically significant alterations are indicated by the asterisk (*).

**Figure 11:**
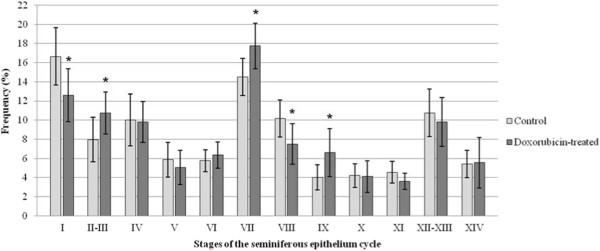
**Frequency of the stages of the seminiferous epithelium cycle in the 64-day-old rats.** At this age, the frequency of the stages I and VIII decreased whereas stages II-III, VII and IX showed an increase of their frequencies. Statistically significant alterations are indicated by the asterisk (*).

**Figure 12:**
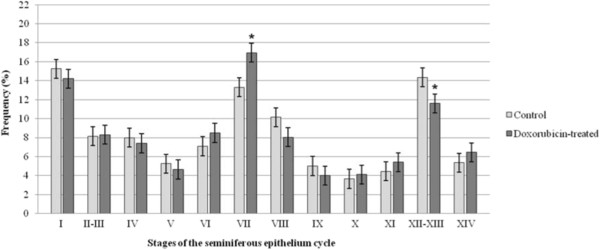
**Frequency of the stages of the seminiferous epithelium cycle in the 127-day-old rats.** At this age, the stage VII showed an increase of its frequency and the stages XII-XII showed a decrease of its frequency. Statistically significant alterations are indicated by the asterisk (*).

The frequency of some stages of the seminiferous epithelium cycle was altered in all doxorubicin-treated rats of each age studied (Figures 10, 11 and 12). At 40dpp, an increase of the frequency of stages I, XI and XIV and a reduction of the frequency of stages II-III, IV, V and VI were observed in the doxorubicin-treated rats (Figure 10) when compared to the corresponding C40 subgroup. In the D64 subgroup, an increase of the frequency of stages II-III, VII and IX and a reduction of the frequency of stages I and VIII were observed when compared with the C64 subgroup (Figure 11). The D127 subgroup showed partial recovery of the synchrony of the seminiferous epithelium cycle. In this subgroup only the frequency of stage VII increased and the frequency of stages XII-XIII decreased when compared with the C127 subgroup (Figure 12).

## Discussion

The deleterious action of doxorubicin on male germ cells has been described [9]. The citotoxity caused by doxorubicin on the seminiferous epithelium can be related to its therapeutic activity; it interferes with molecules associated to the nuclear DNA and with enzymes (RNA and DNA polimerases, topoisomerases I and II) that are active in the cell division process. Then, doxorubicin forms a complex with chromatin [46], blocking the G2 phase of the cell cycle [47,48] and provoking single and/or double strand DNA breaks [49]. Doxorubicin also interferes with membrane lipids [50-53], provoking alterations in their chemical structure and impairing their function. The production of reactive species of oxygen, as a consequence of free radicals caused by doxorubicin, can also affect the cellular functions, altering the cellular metabolism in different aspects [52,54]. In fact, the production of free radicals is one of the factors that limit the therapy with doxorubicin. This anticancer agent produces, as previously mentioned, a significant increase of lipid peroxidation and alterations of antioxidant enzyme activities in different organs of rats, including testis, as observed in vitro [55]. It provokes significant elevation in the testicular malondialdehyde concentrations and decreases of glutathione content, glutathione reductase (GR), glutathione-S-transferase (GST), superoxide dismutase (SOD) and catalase activities, thus indicating oxidative stress production in doxorubicin-induced testicular toxicity [11]. Besides, the deleterious effect of this anticancer agent on adult rat testis lipids and fatty acids has been observed after single or multiple dose regimes, resulting in a gradual loss of spermatogenesis and in a decrease in phospholipids, including glycerophospholipids and sphingomyelin; in this context, glycerophospholipids selectively loose their major polyunsaturated fatty acid (PUFA), while sphingomyelin looses its major very long-chain PUFA (VLCPUFA). By contrast, triglycerides and especially cholesterol esters (CE) tend to accumulate in the testes undergoing germ cell death, probably in the surviving Sertoli cells. Their fatty acid patterns suggest that initially these lipids retained part of the PUFA coming from, or no longer used for the synthesis of germ cell glycerophospholipids. Determining whether this accumulation results from a physiologic adaptation to the effects of doxorubicin or simply reflects another lipid derangement caused by the drug remains to be investigated [56].

Although some of the doxorubicin mechanisms of action on germ cell are known, the effects of this drug on other testicular components are poorly understood. Spermatogonia are the preferential doxorubicin target due to the presence of the enzyme topoisomerase II, but probably primary spermatocytes can also be damaged, although the role of topoisomerase II in pre-mitotic DNA synthesis, at determined stages, is more accentuated than in pre-meiotic synthesis as observed after etoposide-treatment [8]. Thus, DNA synthesis in pre-meiotic spermatocytes is not so vulnerable to the doxorubicin action as pre-mitotic DNA synthesis. Moreover, it is possible that other topoisomerases can be involved in the process of pre-meiotic synthesis.

Thus, considering the different mechanisms of action of doxorubicin previously mentioned, it is also possible that additionally to spermatogonia, other cells, including Sertoli cells, are also targeted by doxorubicin.

In the present study, it was observed that doxorubicin administration to rats at early prepubertal phase alters transferrin production by Sertoli cells at specific phases of testicular development, indicating a functional alteration of these cells. On the other hand, transferrin synthesis by Sertoli cells is dependent on the presence of germ cells [28,30,57]. Thus, it is possible that the diminution of transferrin production by Sertoli cells is a consequence of germ cell depletion caused by doxorubicin. The significant recovery of transferrin synthesis by Sertoli cells and of the seminiferous epithelium observed in D127 subgroups support this hypothesis.

It is important to highlight that doxorubicin reduces the synthesis of the transferrin receptor and leads to atypical changes in intracellular iron distribution and trafficking [58]. In the testes, transferrin receptors are present in the basal region of the Sertoli cell membrane and are essential to promote iron transportation from blood to the germ cells that are localized in the adluminal compartment of the seminiferous epithelium. Iron is crucial for germ cell proliferation and differentiation and Sertoli cells are the only way by which this ion can reach the germ cells at the adluminal compartment. To deliver iron ions to these cells, diferric plasma transferrin is endocytosed through the receptor at the basal region of the Sertoli cell membrane. In the cytoplasm of the Sertoli cells, iron is detached from plasma transferrin, captured by Sertoli cell transferrin and delivered to germ cells [59]. Thus, if transferrin receptors are damaged by doxorubicin, the iron traffic through the seminiferous epithelium could have been affected. In addition, the germ cells localized in the basal compartment of the seminiferous epithelium get iron directly from plasma transferrin [60]. Thus, damages to transferrin receptors could also cause reduction of iron capture by these cells. Indeed, in the D40 subgroup, spermatogonia were negative for transferrin, what may have contributed to the reduction of the volume density of transferrin-positive seminiferous epithelium.

In addition to transferrin receptor alterations, doxorubicin also increases the plasmatic levels of transferrin [61]. Since most of the transferrin present in the interstitial tissue comes from the blood stream, it could be possible that the increase in the volume density of transferrin-labeled interstitial tissue observed in the D64 subgroup was a result of an increase of plasmatic transferrin levels. However, it does not seem to be the case, since the total volume of the interstitial tissue also increased. Moreover, the volume density of transferrin-positive interstitial tissue of D40 and D127 subgroups was normal, indicating that transferrin of this testicular compartment was not affected. This suggests that, in the testis, doxorubicin acted specifically on Sertoli cell transferrin.

Although the damages to Sertoli cell function are generally reversible, the morphological alterations observed after doxorubicin treatment suggest that these cells may have been directly injured in addition to their secondary damage occurred due to the germ cell primary harm. The presence of Sertoli cell nuclei in the tubular lumen, for example, indicates that the structural integrity of these cells was affected by the treatment with doxorubicin. Dislocation of Sertoli cell nuclei from basal to adluminal or luminal compartments has also been demonstrated in adult rats treated with cimetidine [62,63]. In these studies, this alteration was associated to Sertoli cell death. Morevover, in another research, reduction of Sertoli cell number followed by a decrease in sperm production, normal morphology and motility was observed in doxorubicin-treated adult mice, 6 weeks after the end of the treatment [64]. In the present study, although we did not score the Sertoli cell number, the Sertoli cell nuclei detached into the lumen indicate that these cells may have been drastically injured. Thus, besides the secondary Sertoli cell damage, due to the primarily occurred germ cell death, it is also important to consider that when doxorubicin was administered, Sertoli cells were passing through critical phases of their development, what makes a primary damage more likely to occur. Around 15dpp, when the rats received the first dose of doxorubicin, Sertoli cells stop to proliferate [65] and the blood-testis barrier start to be formed [66,67]. At 22dpp, when the second dose was administered, Sertoli cells were still undergoing maturation [65,68]. Therefore, we could also consider the higher susceptibility of Sertoli to doxorubicin at these phases than adult Sertoli cells. Because Sertoli cells are crucial for spermatogenesis, damages in these cells at early pubertal phase could also lead to germ cell death in other later periods of sexual maturation as peripuberty (40 days) and after the completion of puberty (64 days). Hence, considering the Sertoli cell morphological alterations observed in this study, it is possible that the seminiferous epithelium alterations observed in the doxorubicin-treated rats could also be consequence of direct Sertoli cell damage. Indeed, some alterations such as intraepithelial vacuolization, spermatid retention and high frequency of Sertoli cell nuclei in which the nucleolus was not evident suggest that Sertoli cells were damaged independently of germ cell death. Moreover, despite the possibility of the decrease of Sertoli cell transferrin labeling a consequence of germ cell depletion, it is important to consider that doxorubicin can increase the production of free radicals as previously observed [52,54]. In addition, primary immature Sertoli cell obtained from 18-day-old rat testes and cultured with the anticancer agents cis-diamminedichloroplatinum (CDDP), adriamycin and vinblastin revealed that these agents have direct damaging effects on rat Sertoli cell, decreasing the level of transferrin. In this research, the concentration of transferrin in the culture medium was measured and used as an indicative of Sertoli cell function [69]. The role of Sertoli cell in postchemotherapy azoospermia has also been noticed in a 31-year-old patient who underwent cancer cytotoxic chemotherapy for non-Hodgkin's lymphoma at 13 years of age . In this patient, a fraction of Sertoli cells (13%) in the atrophic tubules re-expressed the intermediate CK-18 filament protein, which is normally absent after puberty, but not the D2-40 antigen, a membrane-linked glycoprotein which loss of expression at puberty marks an irreversible step in Sertoli cell maturation. The reversion to a dedifferentiated state, marked by the reexpression of CK-18 as a consequence of chemotherapy, besides the partial inactivation of Sertoli cells following the chemotherapeutic drug cytotoxicity may contribute to the spermatogenic impairment, then resulting in infertility [70]. Although testicular germ cell products can regulate Sertoli cell function [71,72] and alter the production of transferrin, for example, it is also possible that a harmful effect of doxorubicin on Sertoli cell might have occurred in the present study. Disruption of Sertoli cell structure and shedding of immature germ cells have been observed in doxorubicin-treated adult mice [64]. However, other experiments using labeling of house-keeping proteins such as actin and/or markers of Sertoli cell differentiation as cytokeratin-18 must be conducted to better clarify this subject. Another relevant hypothesis is that the blood-testis barrier injury, caused by doxorubicin toxicity, was mediated by the generation of free radicals [1,2] and lipid peroxidation [3]. In fact, studies in the testis and other organs have illustrated the role of environmental toxicant-induced oxidative stress in mediating the disruption of cell junctions, which is regulated by the activation of phosphatidylinositol 3-kinase (PI3K)/c-Src/focal adhesion kinase (FAK) and mitogen-activated protein kinase (MAPK), signaling pathways involving polarity proteins and leading to reproductive dysfunction, such as reduced sperm count and semen quality in men [73]. However, the impact of doxorubicin toxicity on integrity and damage of the blood-testis barrier during prepuberty are still to be established.

Important alterations were observed in the frequency of the seminiferous epithelium cycle after doxorubicin treatment, especially at 64dpp. The seminiferous epithelium cycle is a strictly controlled process that is characterized by specific germ cell associations, defined as stages of the seminiferous epithelium cycle. During this cycle, Sertoli cells change their morphology and function, according to the requirements of the spermatogenic process. Because Sertoli cells are responsible for the synchronization of the seminiferous epithelium cycle, alterations of these cells can cause problems to the progression of the stages during the cycle. It is also important to consider that the massive loss of germ cells disturbs the typical cell association of each stage, leading to alterations of the frequency of the stages of the seminiferous epithelium cycle. Another important factor that should be considered is that postpubertal and adult doxorubicin-treated rats showed retention of step 19 spermatids. At this step, these cells are released into the tubular lumen through a process called spermiation, which occurs at stage VIII of the seminiferous epithelium cycle. This process is controlled by Sertoli cells [74] and injuries to these cells can alter spermiation and cause spermatid retention [75].

Alterations of the seminiferous epithelium cycle has been described after administration of chemicals such as 1, 3 dinitrobenzene [75] and 2, 5 hexanedione [76], which are referred as Sertoli cell toxicants [77,78]. In general, chemotherapeutic drugs are not considered Sertoli cell toxicants. However, previous studies by our group have suggested that etoposide, another chemotherapeutic drug, in addition to causing damage to the germ cells, may also provoke direct damages to Sertoli cells [17,18]. The present study also points to a possible effect of doxorubicin on Sertoli cells. Indeed, some Sertoli cell alterations suggest that these cell damages are more severe than those considered be exclusively secondary effects resulted from germ cell death.

Another important aspect is that the stage-specific gene expression is a fundamental characteristic of rat spermatogenesis and Sertoli cells [79]. In fact, in adult doxorubicin-treated mice, a quantitative RT-PCR analysis showed a dysregulation in the expression of some genes such as *Csk* and *Axl*, which are important to the remodeling of seminiferous tubule during spermatogenesis and to the germ cell differentiation respectively [64]. These remarks could support some of our observations, concerning the conspicuous alterations in the frequency of some seminiferous epithelium stages in doxorubicin-treated rats, in all ages investigated (40, 64 and 127 days). In addition, the inactivation and delay of the Sertoli cell maturation due to cytotoxicity of the chemotherapeutic drugs may contribute to the spermatogenic impairment [70] and could be related to the functional alterations of the Sertoli cell, and probably to the changes in seminiferous epithelium cycle as well. In our report, the delay or interruption of the Sertoli cell differentiation could justify, at least in part, the significant increase of the frequency of stage I and the reduction of subsequent stages such as II -III, IV, V, VI at 40 days, in comparison to the control rats. In addition, it has been shown that the aforementioned stages II and III are infrequently pinpointed as being especially vulnerable to agents that act on spermatogenesis [80]; therefore, it is possible that their frequencies have been altered due to doxorubicin direct action on Sertoli cells. Moreover, as previously mentioned, it is important to remember that doxorubicin was administered during prepubertal phase, when the Sertoli cells were still undergoing maturation.

Summarizing, doxorubicin is a very potent drug that acts through different mechanisms of action. Without doubt, a secondary damage of Sertoli cell occurred due to the injury caused to the germ cells. On the other hand, the alterations observed in the present study, along with the fact that Sertoli cells were not completely mature when doxorubicin was administered, suggest that the direct damage to the Sertoli cell observed is likely to be also responsible, at least in part, for some of the testicular alterations noticed. The iron atypical chelator action of doxorubicin, which provokes the decrease of transferrin receptor synthesis, leading to atypical changes in intracellular iron distribution and trafficking [58], can also alter the synthesis of trasferrin by the Sertoli cell, a phenomenon that should also be considered.

Measurements of transferrin contents in rat testes can indicate damage to Sertoli cell function. High doses of cisplatin (8 mg/kg), for example, affect testicular transferrin concentration, but lower doses (4 mg/kg and 2 mg/kg) have no significant effect on Sertoli cell function. Thus, an anti-cancer agent primarily may affect the DNA synthesizing activity of spermatogonia and spermatocytes, but high doses of these agents have deleterious effects on Sertoli cells [81]. The age of treatment chosen can also be a determining factor in the type of testicular damage observed. However, detailed studies will be necessary to verify the direct damage of Sertoli cell by the anticancer agent doxorubicin when administered in early prepubertal rats.

## Conclusions

The evaluation of harmful action of chemotherapeutic drugs on Sertoli cell during prepuberty is advisable since, at this phase, Sertoli cells play a pivotal role on spermatogenesis, supplying the factors required for germ cell generation and helping to synchronize the development of germ cells at different stages of seminiferous epithelium. These studies can also contribute to a better understanding of the side effects of chemotherapeutic drugs upon the prepubertal testis and upon the male fertility, contributing to the development of chemotherapy protocols intending germ cell protection. Besides, the relationship between the delay in Sertoli cell differentiation or its dedifferentiation with anticancer agents must be scrutinized.

## Competing interests

The authors declare that they have no competing interests.

## Authors' contributions

OB performed all the experimental work. SMM and TS intellectually contributed to the experimental design, results analysis, writing and revision of the manuscript. FKO made the statistical analysis of data. ESC contributed to the final revision of this manuscript. All authors read and approved the final manuscript.
